# Plant species identity and mycorrhizal type explain the root-associated fungal pathogen community assembly of seedlings based on functional traits in a subtropical forest

**DOI:** 10.3389/fpls.2023.1251934

**Published:** 2023-10-27

**Authors:** Yongning Li, Yan Xie, Zhengjiao Liu, Liuqing Shi, Xubing Liu, Minxia Liang, Shixiao Yu

**Affiliations:** State Key Laboratory of Biocontrol, School of Life Sciences, Sun Yat-sen University, Guangzhou, China

**Keywords:** abiotic environments, community assembly, functional traits, mycorrhizal type, neighborhood plants, root-associated fungal pathogens, species identity

## Abstract

**Introduction:**

As a crucial factor in determining ecosystem functioning, interaction between plants and soil-borne fungal pathogens deserves considerable attention. However, little attention has been paid into the determinants of root-associated fungal pathogens in subtropical seedlings, especially the influence of different mycorrhizal plants.

**Methods:**

Using high-throughput sequencing techniques, we analyzed the root-associated fungal pathogen community for 19 subtropical forest species, including 10 ectomycorrhizal plants and 9 arbuscular mycorrhizal plants. We identified the roles of different factors in determining the root-associated fungal pathogen community. Further, we identified the community assembly process at species and mycorrhizal level and managed to reveal the drivers underlying the community assembly.

**Results:**

We found that plant species identity, plant habitat, and plant mycorrhizal type accounted for the variations in fungal pathogen community composition, with species identity and mycorrhizal type showing dominant effects. The relative importance of different community assembly processes, mainly, homogeneous selection and drift, varied with plant species identity. Interestingly, functional traits associated with acquisitive resource-use strategy tended to promote the relative importance of homogeneous selection, while traits associated with conservative resource-use strategy showed converse effect. Drift showed the opposite relationships with functional traits compared with homogeneous selection. Notably, the relative importance of different community assembly processes was not structured by plant phylogeny. Drift was stronger in the pathogen community for ectomycorrhizal plants with more conservative traits, suggesting the predominant role of stochastic gain and loss in the community assembly.

**Discussion:**

Our work demonstrates the determinants of root-associated fungal pathogens, addressing the important roles of plant species identity and plant mycorrhizal type. Furthermore, we explored the community assembly mechanisms of root-associated pathogens and stressed the determinant roles of functional traits, especially leaf phosphorus content (LP), root nitrogen content (RN) and root tissue density (RTD), at species and mycorrhizal type levels, offering new perspectives on the microbial dynamics underlying ecosystem functioning.

## 1 Introduction

Based on the premise that a natural enemy has restricted ability to disperse (Adler and Muller-Landau, 2005) and a certain degree of specificity (Sedio and Ostling, 2013), fungal pathogens could result in conspecific negative density dependence (CNDD) on seedlings, facilitating the coexistence of plant species (Janzen, 1970; Connell, 1971). Root-associated fungi serve as an effective indicator of plant-fungus network, which has been considered to mediate the diversity maintenance and population dynamics(Tedersoo et al., 2020; Kuang et al., 2021). However, the community structure and assembly processes of root-associated fungal pathogens belonging to forest seedlings remains poorly understood. Given this, understanding the properties and drivers of root-associated fungal pathogens is important for better understanding of the forest dynamics (Trivedi et al., 2017; Zitnick-Anderson et al., 2020; Yang et al., 2021).

Both biotic and abiotic factors influence the composition of soil-borne fungal communities. The important role of species identity has been stressed in various studies (Morris et al., 2009; Liu et al., 2021; Sweeney et al., 2021). Notably, the influence of plant species identity on fungal community could be modulated by plant functional groups (Sweeney et al., 2021). Abiotic environments (Tedersoo et al., 2016) and neighboring plants (Bahram et al., 2013; Morris et al., 2013; Chen et al., 2018) are also believed to be important drivers of community composition. Plant species identity tended to dominate at local scale (Leff et al., 2018) while plant community composition seemed more important at larger scale (Prober et al., 2015) in terms of soil fungal community. However, there are few studies concerning the community determinants of root-associated fungal pathogens that regulate ecosystem functioning (Bever et al., 2015). Revealing the relative importance of biotic and abiotic factors for such root-associated fungal pathogen communities could contribute to filling the research gap in belowground fungal communities.

As mediators of plant interactions with pathogens, mycorrhizal fungi play a crucial role in plant productivity and community dynamics. There are two major mycorrhizal types in the study site Heishiding Nature Reserve, including arbuscular mycorrhiza (AM) and ectomycorrhiza (ECM). Arbuscular mycorrhiza exist in nearly 80% of plant species and tend to experience more negative feedback resulting from soil biota (Kadowaki et al., 2018). In comparison, ectomycorrhizal plants show positive density dependence in temperate and subtropical forests (Chen et al., 2019a; Sasaki et al., 2019), probably due to more effective resistance to pathogens (Tedersoo et al., 2020). Though many studies have confirmed the evolutionary and functional differences of these two major mycorrhizal types (Brundrett and Tedersoo, 2018; Kadowaki et al., 2018), few studies have explored the effects of mycorrhizal type on host-associated microbiome (Bahram et al., 2020; Liang et al., 2020), especially root-associated pathogens.

Two processes simultaneously function in the formation of microbial communities, namely, deterministic and stochastic processes (Ofiteru et al., 2010; Stegen et al., 2016). Deterministic processes imply that deterministic factors, such as species traits and environmental conditions, play a central role in community structure (Chesson, 2000). Conversely, stochastic processes stress the importance of birth, death, colonization, extinction, and speciation (Chave, 2004). Currently, their relative contribution to the formation of the microbial community is at the forefront of research (Gao et al., 2020; Huo et al., 2023; Jiao et al., 2023).

Many studies have confirmed the potential drivers of the fungal community assembly process (Gao et al., 2020; Wang et al., 2022; Zheng et al., 2022), stressing the importance of global change and plant traits. However, less attention has been paid into the root-associated fungal pathogen community. Elucidating the assembly processes and underlying drivers of root-associated fungal pathogen community could help reveal the belowground microbial dynamics in the forests. Besides, understanding the effects of mycorrhizal type on root-associated pathogen community assembly, which could differentiate in alleviating negative density dependence (Tedersoo et al., 2020), would provide essential insights into the mutualistic relationships in the forest community.

In this study, we examined the impacts of abiotic and biotic factors, namely plant species identity, plant habitat, and plant mycorrhizal type, on the composition of the root-associated fungal pathogen community. We hypothesized that species identity and mycorrhizal type play more important roles on the fungal pathogen community than habitat conditions. Regarding the pathogen assembly mechanisms, we hypothesized that the relative importance of different community assembly process should vary with plant species identity and could be explained by the plant functional traits or plant phylogeny. The comparison of different community assembly process between AM plants and ECM plants should be in consistence with the findings at species level.

## 2 Materials and methods

### 2.1 Study site

The field study was carried out within a 50-ha forest at Heishiding Nature Reserve (Liang et al., 2019; Wang et al., 2019; Li et al., 2021) in Southern China (centered on 111°53′E, 23°27′N), which supports the growth of more than 1600 seed plant species belonging to 669 genera and 188 families. The annual mean precipitation is about 1744 mm and the annual mean temperature is about 19.6°C. In 2011, a stem-mapping survey was conducted on the 50-ha plot to map trees and shrubs with a diameter at breast height (DBH) ≥ 1 cm, resulting in more than 269,000 individual stems.

### 2.2 Field survey and molecular characterization

Nineteen tree species were selected as the focal species (**Table S1**), including 9 arbuscular mycorrhizal trees and 10 ectomycorrhizal trees (Brundrett, 2009). Seedlings with size between 10-30 cm of the same species were at least 30 m apart from each other, with different species at least 5 m apart. Root system belonging to a specific seedling was dug out to collect the root samples. In total, 250 root samples were collected. Root samples were taken from the fine root, and the surface was sterilized (1 min 75% ethanol, 3 min 2.63% NaClO, 1 min 75% ethanol, 1 min distilled water). Total genomic DNA from each root sample was extracted using the cetyl trimethylammonium bromide (CTAB) protocol (Allen et al., 2006). Using the tagged fungal specific primers ITS1-1F forward (5’-CTTGGTCATTTAGAGGAAGTAA-3’) and ITS1-1F reverse (5’-GCTGCGTTCTTCATCGATGC-3’) (White et al., 1990), the fragments of the internal transcribed spacer (ITS) were amplified by polymerase chain reaction (PCR). The PCRs were performed in a 50-μL reaction mixture containing 25 μL PhusionMasterMix 2× (Phusion^®^ High-Fidelity PCR Master Mix with GC Buffer), 1 μL of each primer (Primer F and Primer R), 2~10 μL gDNA, and ddH_2_O. The PCR amplification profile was set as follows: initial denaturation of 5 min at 95°C, followed by 34 cycles of 1 min at 94°C, 45 s at 57°C, and 1 min at 72°C. Then 10 min at 72°C and 5 min at 16°C were added as final additional extensions. The PCR products were detected by 2% agarose gel electrophoresis and purified with a GeneJET DNA gel extraction kit (Thermo Scientific, USA). The amplicon library was conducted by Ion Plus Fragment Library Kit 48 rxns, passing Qubit quantification and library detection. Later, the amplicon library was sequenced on Ion S5™ XL Ion 530 Chip system (Thermo Fisher, USA).

Cutadapt V1.9.1 was used to remove low-quality reads, barcodes, primers, and chimera, resulting in 77740 clean reads on average. Based on a 97% similarity cutoff, we clustered the operational taxonomic units (OTUs) from the root samples using UPARSE v7.0.1001. All OTU sequences were annotated by QIIME 1.9.1 against the UNITE database. The current sequencing depth is sufficient to reflect the microbial diversity contained in the samples based on rarefaction curve. We analyzed the community composition at the species level, assigning all fungal species into functional guilds by the FUNGuild algorithm and database (Nguyen et al., 2016). Fungal species assigned as plant pathogens only were retained, resulting in 86 putative fungal pathogens. Notably, fungal pathogens accounted for 9.40% of the fungal community. Referring to a fungal phylogeny based on 18S + 28S rDNA sequences, the phylogenetic tree for pathogens was constructed with the taxonomy_to_tree.pl script (Tedersoo et al., 2018).

### 2.3 Functional traits and plant phylogeny

Sixteen species-level functional traits of the seedlings were measured in the forest (Shen et al., 2019): leaf area (LA, cm^2^), leaf area ratio (LAR, cm^2^ g^−1^), leaf carbon content (LC,%), leaf dry matter content (LDMC, g g^−1^), leaf nitrogen content (LN, g kg^−1^), leaf phosphorus content (LP, g kg^−1^), specific leaf area (SLA, cm^2^ g^−1^), specific stem length (SSL, cm g^−1^), leaf thickness (T, cm), fine-root diameter (DIAM, mm), root branching intensity (RBI, tips), root nitrogen content (RN, g kg^−1^), root phosphorus content (RP, g kg^−1^), root tissue density (RTD, g cm^−3^), specific root area (SRA, cm^2^ g^−1^), and specific root length (SRL, cm g^−1^). A phylogenetic tree of the focal species was constructed based on ‘V.PhyloMaker’ package (Jin and Qian, 2019).

### 2.4 Environmental variables and neighboring plants

Environmental variables for the focal seedlings included four topographic variables and twenty-six edaphic variables. These data were obtained from the Heishiding Database (Luo et al., 2021) through Kriging interpolation. The indicators describing neighboring plants for focal seedlings included the conspecific and heterospecific basal area of adult trees within 5 m from the seedlings. Due to vague orientation, the locations for focal seedlings were represented by the nearby focal adult tree or different adult tree (in the absence of a focal adult tree), with a roughly 2-m deviation.

### 2.5 Statistical analysis

To evaluate the effects of plant species identity, plant habitat, and plant mycorrhizal type in determining the root-associated fungal pathogen community, we performed permutational multivariate analysis of variance (PERMANOVA) based on the ‘adonis’ function in the vegan package (Dixon, 2003). In terms of plant habitat, abiotic environments showing significant effects when tested separately were selected for PERMANOVA analysis to determine the main factors. Differences (statistic R-values) in the root-associated fungal pathogen community between the focal species were measured by analysis of similarities (ANOSIM) based on pairwise tests. The non-randomness of root-associated fungal pathogen community was tested by calculating H2′ (Blüthgen et al., 2006). Based on potential associations, H2′ values ranged from 0 (indicating absence of network specialization) to 1 (indicating complete network specialization). The calculation of H2’ was performed using the H2fun function within the bipartite package (Dormann et al., 2009). Based on partial least square-discriminant analysis (PLS-DA) and ANOSIM, we evaluated the effect of different mycorrhizal plants on root-associated fungal pathogens. The contributions of deterministic (heterogeneous selection and homogeneous selection) and stochastic processes (dispersal limitation, homogenizing dispersal and drift) on pathogen communities were also inferred by phylogenetic bin-based null model analysis (iCAMP) (Ning et al., 2020), basically including the beta Net Relatedness Index (βNRI) and modified Raup-Crick metric (RC). Based on the modified stochastic processes calculated by pNST, we set the bin size limit as 10. We detect phylogenetic signal within community assembly processes based on the ‘phyloSignal’ function in the ‘phylosignal’ package (Keck et al., 2016) to test the effect of plant phylogeny. We also further determined the effects of different functional traits on community assembly process based on linear regression model at species level. Sixteen tree species were selected because complete trait data were available for them. The differences of community assembly process at mycorrhizal type level were also identified and the significance was calculated by bootstrapping for 1000 times. We compared functional traits between arbuscular mycorrhizal plants and ectomycorrhizal plants using t.test. All analyses were conducted in R (R Development Core Team, 2017).

## 3 Results

### 3.1 Influence of different factors on the root-associated fungal pathogen

**Figure 1:**
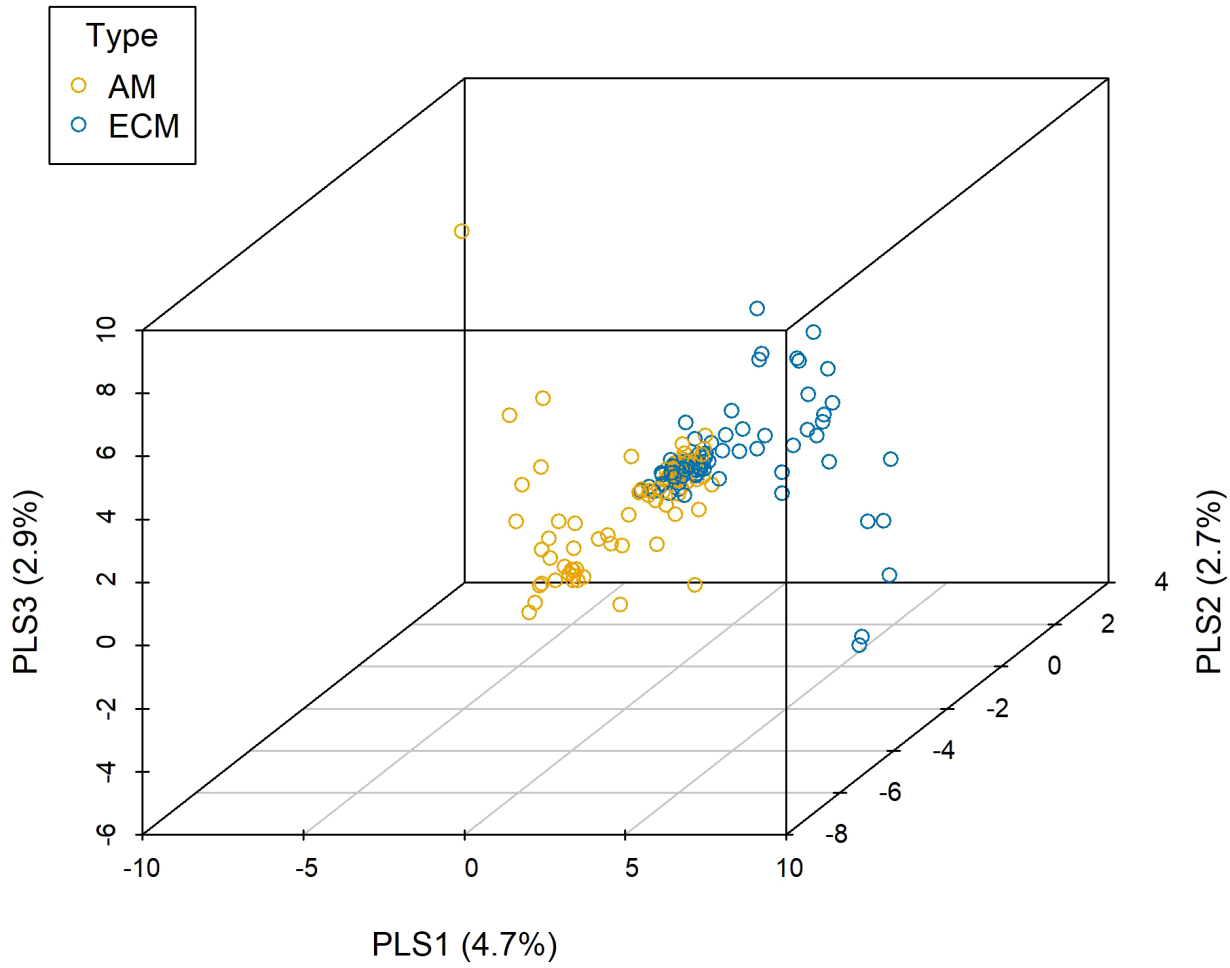
Partial least square-discriminant analysis (PLS-DA) for fungal pathogens associated with different mycorrhizal plants. AM, fungal pathogens associated with arbuscular mycorrhizal trees; ECM, fungal pathogens associated with ectomycorrhizal trees.

Abiotic environment, neighboring plants, mycorrhizal type, and species identity all determined the root-associated fungal pathogen community based on the results from PERMANOVA (**Table 1**), in which species identity had a major role. Five new variables representing the abiotic environments were extracted through principal components analysis, accounting for 52.49% of the total variation. We selected the environmental factors showing significant effects when tested separately (**Table S2**) to perform the PERMANOVA, and the results showed that pH, SOM, TP, AP, and convexity were the key determinants in terms of abiotic environment (**Table S3**). This implied that the characterizations of the abiotic environment were well represented by the common edaphic physicochemical properties. In terms of neighboring plants, a significant effect was detected for conspecific basal area rather than heterospecific basal area. The observed network-level specialization index for the root-associated fungal pathogen community was 0.50, while the null model analysis obtained 0.27, revealing that the observed network specialization was significantly higher than expected by chance (**Table S4**) and stressing the importance of species identity. Based on PLS-DA (**Figure 1**, 10.2% variations explained totally), we found that different mycorrhizal plants showed specific preferences for root-associated fungal pathogens (analysis of similarity [ANOSIM]: R = 0.037, *P* = 0.002), which is in accordance with the PERMANOVA analysis (F = 2.097, df = 1, *P* = 0.002, full model).

**Table 1:** Relative importance of abiotic environment, neighborhood plants, mycorrhizal type and species identity on root-associated fungal pathogen community based on PERMANOVA.

	r2	p	model
**env_pc1**	**0.007**	**0.024**	abiotic environment+ plant species identity
**env_pc2**	**0.009**	**0.001**	
**env_pc3**	**0.006**	**0.033**	
**env_pc4**	**0.006**	**0.043**	
env_pc5	0.004	0.361	
**host**	**0.105**	**<0.001**	
**con**	**0.007**	**0.014**	neighborhood plants+ plant species identity
hetero	0.005	0.215	
**host**	**0.108**	**<0.001**	
**myco_type**	**0.011**	**<0.001**	mycorrhizal type+ plant species identity
**host**	**0.101**	**<0.001**	
**env_pc1**	**0.007**	**0.024**	abiotic environment+ neighborhood plants+ mycorrhizal type+ plant species identity
**env_pc2**	**0.009**	**0.001**	
**env_pc3**	**0.006**	**0.021**	
**env_pc4**	**0.006**	**0.038**	
env_pc5	0.004	0.358	
con	0.005	0.145	
hetero	0.005	0.182	
**myco_type**	**0.009**	**<0.001**	
**host**	**0.094**	**<0.001**	

env_pc1, env_pc2, env_pc3, env_pc4, env_pc5, the first five principal components for the abiotic environments; con, conspecific basal area; hetero, heterospecific basal area. Significant effects are indicated with the bold values.

### 3.2 Influence of functional traits on the root-associated fungal pathogen community assembly

**Figure 2:**
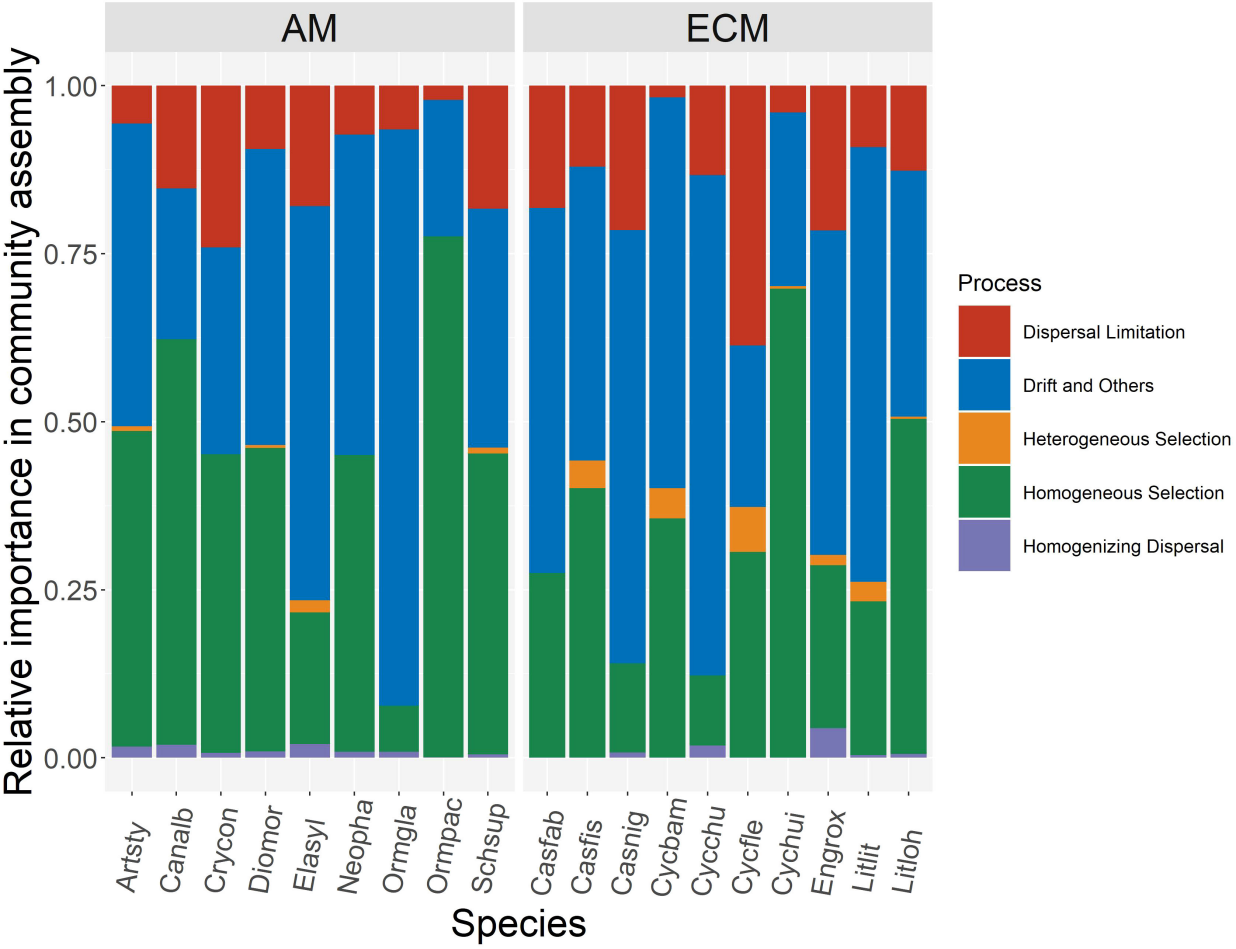
Relative importance of different community assembly process in the root-associated fungal pathogen community of nineteen species. Artsty, *Artocarpus styracifolius*; Canalb, *Canarium album*; Casfab, *Castanopsis fabri*; Casnig, *Castanopsis nigrescens*; Crycon, *Cryptocarya concinna*; Cycbam, *Cyclobalanopsis bambusaefolia*; Cycchu, *Cyclobalanopsis chungii*; Cycfle, *Cyclobalanopsis fleuryi*; Cychui, *Cyclobalanopsis hui*; Diomor, *Diospyros morrisiana*; Elasyl, *Elaeocarpus sylvestris*; Engrox, *Engelhardtia roxburghiana;* Litlit, *Lithocarpus litseifolius*; Litloh, *Lithocgarpus lohangwu*; Neopha, *Neolitsea phanerophlebia*; Ormgla, *Ormosia glaberrima*; Ormpac, *Ormosia pachycarpa*; Schsup, *Schima superba*.

**Figure 3:**
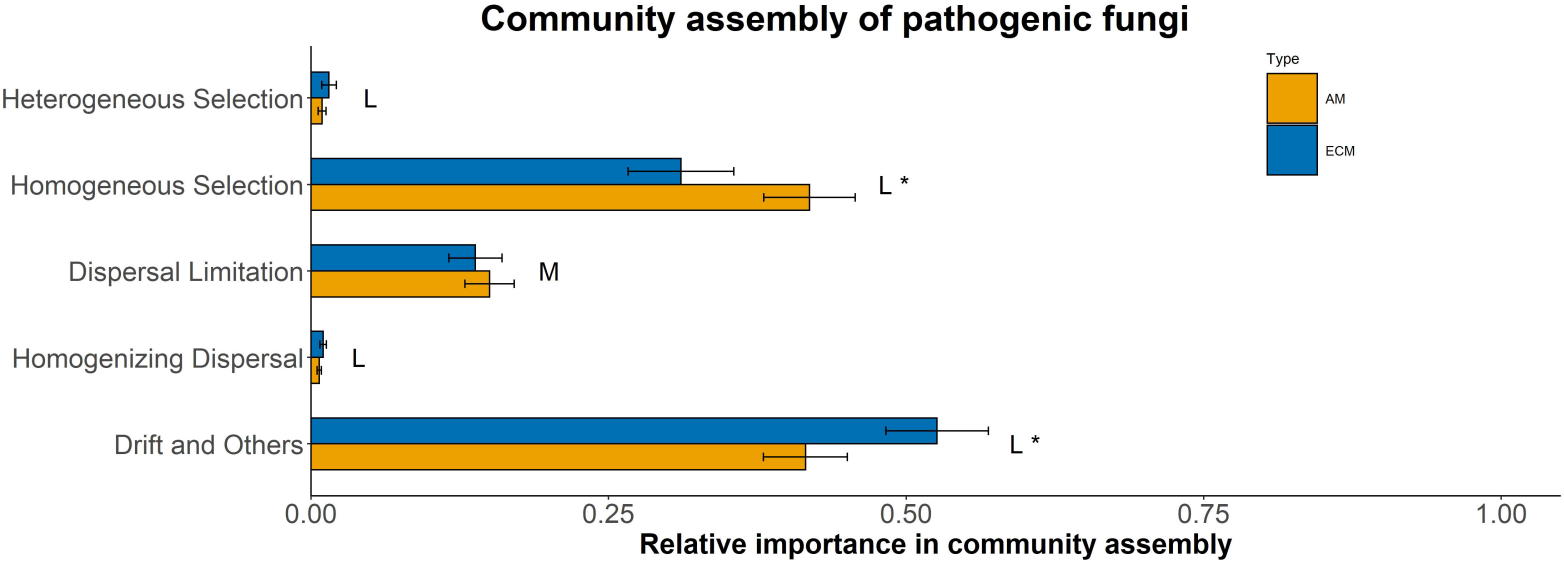
Relative importance of assembly processes in shaping fungal pathogen communities in AM and ECM plants based on iCAMP. One-side significance was measured based on bootstrapping test. L and M represented large (|d| > 0.8) and medium (0.5 < |d| ≤ 0.8) effect sizes based on Cohen’s d. *P < 0.05.

**Figure 4:**
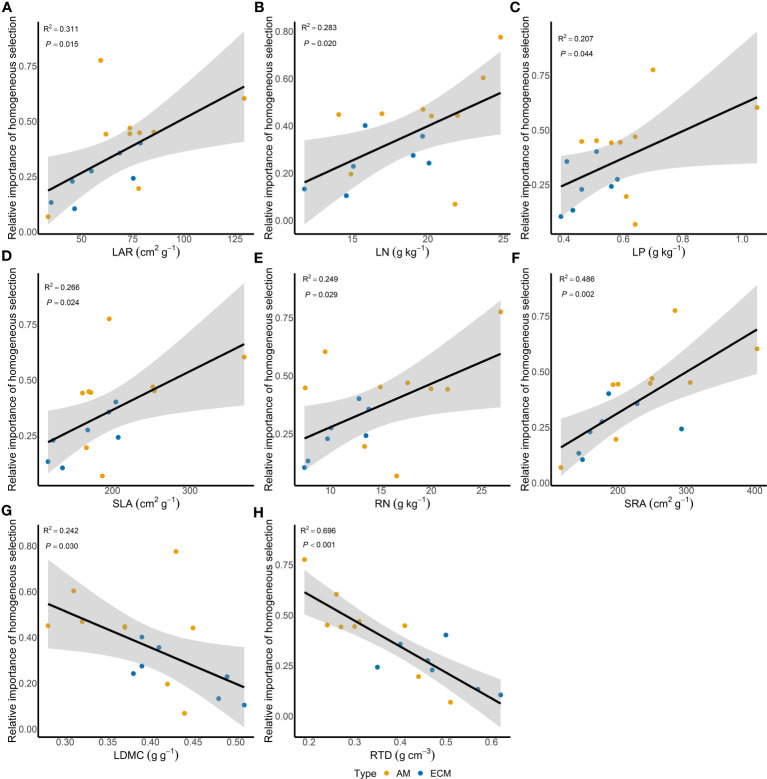
Effects of different functional traits on the relative importance of homogeneous selection. LAR, LN, LP, SLA, RN and SRA are functional traits indicative of acquisitive resource-use strategy **(A–F)**, while LDMC and RTD are functional traits indicative of conservative resource-use strategy **(G–H)**. Solid lines represent regression lines with significant effect, and shaded areas represent 95% confidence intervals. LAR, leaf area ratio; LN, leaf nitrogen content; LP, leaf phosphorus content; SLA, specific leaf area; RN, root nitrogen content; SRA, specific root area; LDMC, leaf dry matter content; RTD, root tissue density.

**Figure 5:**
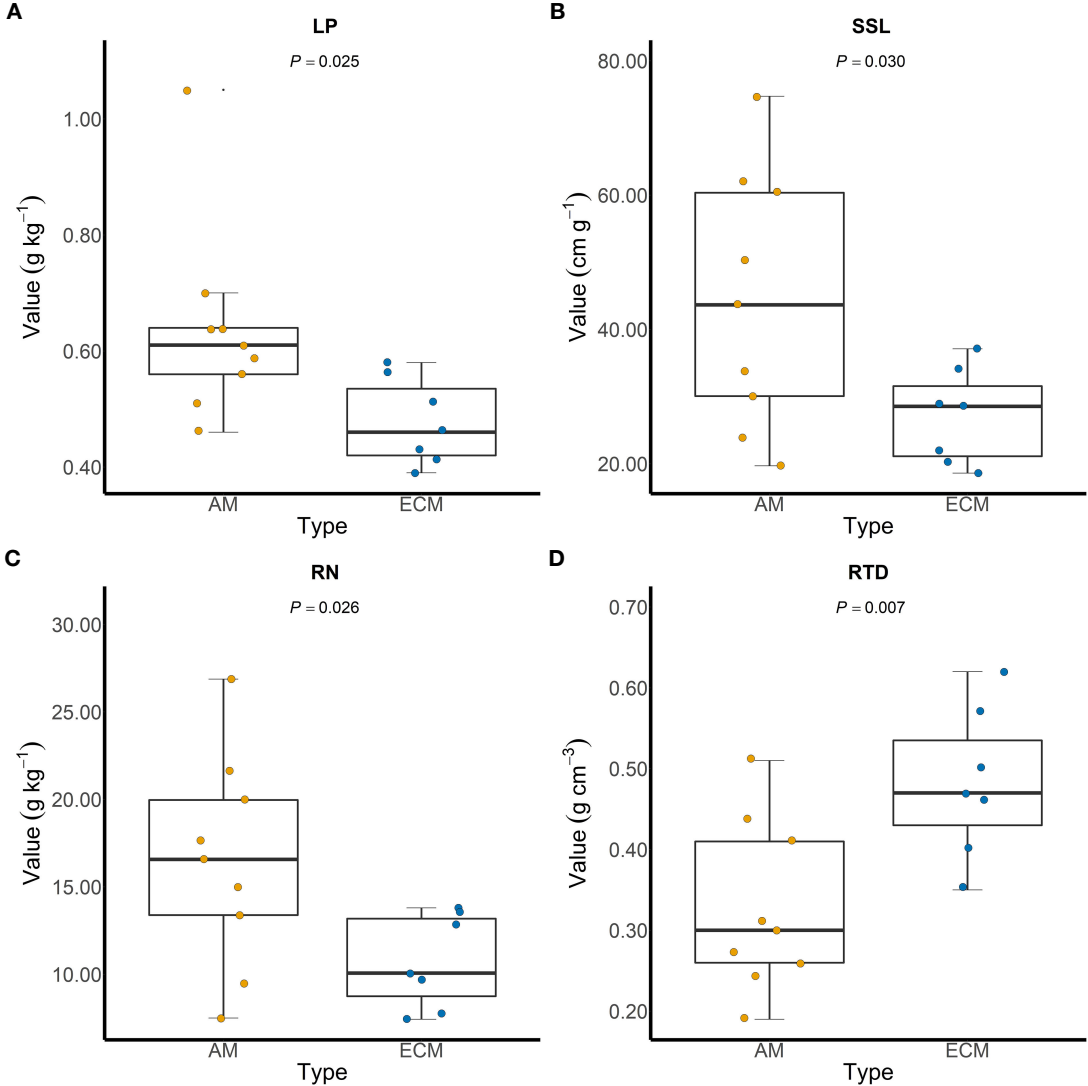
Comparisons of functional traits between arbuscular mycorrhizal plants and ectomycorrhizal plants. LP, SSL and RN are functional traits indicative of acquisitive resource-use strategy **(A–C)**, while RTD is indicative of conservative resource-use strategy **(D)**. LP, leaf phosphorus content; SSL, specific stem length; RN, root nitrogen content; RTD, root tissue density.

Based on iCAMP, we found that the relative importance of five assembly processes, namely heterogeneous selection, homogeneous selection, dispersal limitation, homogenizing dispersal, and drift, varied with the plant species identity. Homogeneous selection and drift played the dominant roles in the community assembly (**Figure 2**). Further, the importance of different processes in the fungal pathogen assembly in AM and ECM plant roots was evaluated. Drift showed greater importance in shaping fungal pathogens in ECM plant species while homogeneous selection dominated in AM species (**Figure 3**). Such results suggest that it is the stochastic processes underlying the loss and gain of fungi, such as stochastic births and deaths, that critically shape the community assembly of root-associated fungal pathogens for ECM plant species. Notably, we found that the relative importance of homogeneous selection and drift were not structured by the plant phylogeny (**Figure S2**). We further determined the effects of different functional traits on such ecological processes with linear regression. Interestingly, the relative importance of homogeneous selection was positively correlated with the functional traits that indicating acquisitive resource-use strategy, namely LAR, LN, LP, SLA, RN and SRA (**Figures 4A–F**), while negatively correlated with the traits indicative of conservative resource-use strategy, including LDMC and RTD (**Figures 4G, H**). Notably, the significant relationships between leaf traits and community assembly process stressed the coordinated effects of leaf and root traits. On the contrary, we found relationships between functional traits and drift on the opposite direction compared with homogeneous selection. LAR, LP, SLA, SRL and SRA (**Figures S1A–E**), which associate with acquisitive resource-use strategy, tended to decrease the relative importance of drift in the community assembly, while LDMC and RTD (**Figures S1F, G**) tended to promote the influence of drift on the community assembly. The effects of other functional traits are detailed in **Table S5**. Based on the comparisons of functional traits between AM plants and ECM plants, we found that the traits indicative of acquisitive resource-use strategy, namely LP, SSL and RN, were higher for AM plants than ECM plants (**Figures 5A–C**). Conversely, higher RTD was found for ECM plants than AM plants (**Figure 5D**). Such results were in line with the findings based on species level. In summary, we identified the relative importance of homogeneous selection and drift driven by functional traits on the root-associated fungal pathogen community assembly, highlighting either the positive effects imposed by acquisitive functional traits or the negative effects imposed by conservative functional traits on homogeneous selection and the converse effect on drift.

## 4 Discussion

The present study unveiled the effect of different factors on the root-associated fungal pathogen community composition, stressing the importance of plant species identity, plant habitat, and plant mycorrhizal type. In terms of the relative importance of community assembly process, species identity and plant mycorrhizal type could also explain the variations based on functional traits.

We focused on abiotic environments and neighboring plants to explore the effects of plant habitat. In terms of abiotic environments, many studies have confirmed the corresponding effect in root-associated fungi (Yu et al., 2013; Blaalid et al., 2014; Zhong et al., 2018), and our results showed that pH, SOM, TP, AP, and convexity had significant effects on root fungal pathogen community. Soil pH and organic matter are considered as two main factors in driving belowground microbiome (Guo and Gong, 2014; Montiel-Rozas et al., 2017; Ballauff et al., 2021), and our study confirmed such widespread effects on the root-associated fungal pathogens. Regarded as the nutrient that most strongly limit the plant growth in the subtropical forest (Condit et al., 2013; Liu et al., 2018), soil phosphorous was also testified to impose significant effect on the root-associated fungi in our study. Available phosphorous also functions in plant immunity (Chan et al., 2021), which directly affects plant susceptibility to different pathogens. The potential role of convexity suggests the importance of topography when considering the factors affecting the belowground microbiome, which has been demonstrated for soil microbial activity and composition (Taş et al., 2018; Chen et al., 2019b; Fairbanks et al., 2020). The plant neighborhood is a great indicator of soil fungal composition, including pathogenic or mycorrhizal fungi (Hubert and Gehring, 2008; Hantsch et al., 2014; Chen et al., 2018), and was confirmed as a significant determinant of root-associated fungal pathogens in our study, in which the basal area of conspecific adults played a major role. Based on the detailed dataset and comprehensive analysis, we emphasized that host habitat, including abiotic environments and neighboring plants, could explain root-associated fungal pathogens to some extent.

Plant species identity has been demonstrated to play a significant role in root-associated fungal community when considering plant habitat or plant mycorrhizal type, indicating its dominant role among various determinants (Botnen et al., 2020; Francioli et al., 2020). Based on the plant–pathogen interaction network, our findings confirmed host specificity for the root fungal pathogen community, which aligns with the finding from prior research (Cheng and Yu, 2020). Understanding the mechanisms underlying community diversity is a central topic in ecology, especially in microbial ecology (Zhou and Ning, 2017). Under the framework that infers community assembly mechanisms by phylogenetic bin-based null model analysis (Ning et al., 2020), we quantify the relative importance of different community assembly process of the root-associated fungal pathogen community of nineteen species. Ecological (Vivanco and Austin, 2008) and evolutionary (Botnen et al., 2020) processes may be related to species identity, in which plant functional traits (Leff et al., 2018) and plant phylogeny (Barberan et al., 2015) serve as important indicators. Our results showed that rather than plant phylogeny, functional traits mediated the community assembly process. Traits associated with acquisitive resource-use strategy promoted homogeneous selection and weaken the effect of drift, while opposite direction was detected for traits associated with conservative resource-use strategy. Using comprehensive traits indexes, our findings supplemented the available knowledge on the relationships between plant traits and root-associated fungal pathogen community assembly, indicating combined effects from aboveground and belowground functional traits on root-associated fungal pathogens. In summary, our results indicated that plant traits rather than plant phylogeny could explain the effect of plant species identity on root-associated fungal pathogen community assembly.

Owing to their association with plant nutrient acquisition strategies, mycorrhizal associations also influence plant resistance to soil-borne pathogens (Tedersoo et al., 2020). In our study, plant mycorrhizal type accounted for the variations in root-associated fungal pathogen community, indicating host specificity for fungal pathogens at the mycorrhizal-type level. Many studies have stressed the effects of plant functional groups on soil-borne fungi (Davison et al., 2020; Francioli et al., 2021; Sweeney et al., 2021), while less attention has been paid to the influence of plant mycorrhizal type, which also implies distinct functionalities in terms of habitat modification (Tedersoo et al., 2020). In accordance with previous researches (Valverde-Barrantes et al., 2018; De La Riva et al., 2021), our findings showed that ECM plants were associated with more conservative traits than AM plants, that is, lower LP, SSL and RN while higher RTD. Consistent with the relationship between functional traits and community assembly based on species level, stronger effect of drift and weaker homogeneous selection was detected in the root-associated fungal community assembly of ECM plants. This could also possibly be explained by the differences in mycorrhizal structure. Mycelium mantles are formed by ECM fungi surrounding the tips of roots (Agerer, 1991), whereas AM fungi stimulate the deposition of root callose around infected root cells (Pozo et al., 2002), which results in a greater physical barrier in the fine root of ECM plants. To be noted, weaker homogeneous selection or stronger drift could indicate the pathogen community with less similar composition, likely facilitating the escape from negative density dependence. Overall, our results provide new insights into the belowground microbial dynamics for different mycorrhizal plants, which associated with the differences in negative density dependence.

Notably, functional classification of fungi through FUNGuild database (Nguyen et al., 2016) could present potential problem caused by the DNA sequences-based prediction and the annotations at larger taxon. Despite the deficiency, the database has been widely adopted for functional classification(Chen et al., 2019a; Delgado-Baquerizo et al., 2020; Liu et al., 2021). Subsequently, the actual pathogenicity of the potential fungal pathogen is highly dependent on the context, that is, the presence of other surrounding microbes (Malik et al., 2016; Liu et al., 2023) and the environmental changes (Liu and He, 2019; Tannous et al., 2020) could lead to variations in the pathogenicity. Future research should focus on how the context dependence could affect the association between fungal pathogen community and the actual pathogenicity, to provide more comprehensive insights into the interaction between forest plants and fungal pathogens.

In conclusion, we explored the root-associated fungal pathogen community using high-throughput sequencing technology. Taking abiotic and biotic factors into account, we identified the roles of plant species identity, plant habitat, and plant mycorrhizal type in shaping the fungal pathogen community composition. Furthermore, we quantified the relative importance of different community assembly of the pathogen community at either species or mycorrhizal type level and highlighted the determinant roles of functional traits. Our findings also implied that the relative importance of homogeneous selection and drift could indicate negative density dependence, promoting our understanding of the belowground microbial dynamics that greatly impact forest community structure and ecosystem functioning.

## Data availability statement

The raw data supporting the conclusions of this article will be made available by the authors, without undue reservation.

## Author contributions

YL, SY designed the study. YL and YX collected the data in the field with assistance from ZL and LS. YL performed statistical analyses. YL wrote the first draft and others contributed to revisions. All authors contributed to the article and approved the submitted version.
