# Supplementary material for: Plant species identity and mycorrhizal type explain the root-associated fungal pathogen community assembly of seedlings based on functional traits in a subtropical forest
Source: Front Plant Sci. 2023 Oct 27;14:1251934. doi: 10.3389/fpls.2023.1251934 (PMC10641815; doi:10.3389/fpls.2023.1251934)
Supplement: Supplementary file 1 [file DataSheet_1.pdf]

Table S1 Focal species and the sample replicates in the study.

**Scientific name**	**Type**	**Replicates**
Castanopsis nigrescens	ECM	18
Ormosia pachycarpa	AM	17
Diospyros morrisiana	AM	15
Schima superba	AM	15
Neolitsea phanerophlebia	AM	15
Elaeocarpus sylvestris	AM	15
Lithocarpus litseifolius	ECM	15
Cryptocarya concinna	AM	15
Artocarpus styracifolius	AM	15
Lithocarpus lohangwu	ECM	15
Cyclobalanopsis chungii	ECM	13
Engelhardtia roxburghiana	ECM	13
Canarium album	AM	12
Cyclobalanopsis bambusaefolia	ECM	11
Ormosia glaberrima	AM	11
Cyclobalanopsis hui	ECM	11
Castanopsis fabri	ECM	10
Castanopsis fissa	ECM	10
Cyclobalanopsis fleuryi	ECM	8

Table S2 Effects of different environmental environments on root-associated fungal pathogen community tested separately.

	R^2	*P*
pH	0.008	0.002
SOM	0.008	0.007
TN	0.007	0.027
TP	0.009	<0.001
AP	0.009	0.003
AF	0.008	0.004
TEB	0.007	0.031
EH	0.01	<0.001
EAl	0.008	0.003
ALT	0.009	0.002
Convexity	0.008	0.004

SOM, soil organic matters; TN, total nitrogen; TP, total phosphorous; AP, available phosphorous; AF, available iron; TEB, total exchangeable base; EH, exchangeable hydrogen; EAl, exchangeable aluminum; ALT, altitude.

Table S3 Effects of different abiotic environments on root-associated fungal pathogen community based on PERMANOVA.

	R^2	*P*
**pH**	**0.008**	**0.002**
**SOM**	**0.008**	**<0.001**
TN	0.004	0.382
**TP**	**0.007**	**0.017**
**AP**	**0.007**	**0.022**
AF	0.006	0.076
TEB	0.004	0.400
EH	0.004	0.576
EAl	0.003	0.803
ALT	0.006	0.085
**convexity**	**0.007**	**0.033**

SOM, soil organic matters; TN, total nitrogen; TP, total phosphorous; AP, available phosphorous; AF, available iron; TEB, total exchangeable base; EH, exchangeable hydrogen; EAl, exchangeable aluminum; ALT, altitude.

Table S4 Result of the network-level specialization index H2’ and t-test with Null model.

	Observed H2’	Null model H2’	t test	*p*
Fungal pathogen community	0.50	0.27	-477.56	<0.001

Table S5 Relationships between functional traits and fungal pathogen community assembly process based on linear regression.

Trait	Homogeneous Selection		Drift		Dispersal Limitation	
	P	r	*P*	r	*P*	r
LA	0.796	0.070	0.601	-0.142	0.754	0.085
LAR	0.015	0.597	0.002	-0.712	0.620	0.134
LC	0.796	-0.070	0.961	0.013	0.450	0.203
LDMC	0.030	-0.541	0.013	0.603	0.831	-0.058
LN	0.020	0.575	0.072	-0.462	0.207	-0.333
LP	0.044	0.510	0.038	-0.522	0.970	-0.010
SLA	0.024	0.562	0.029	-0.546	0.575	-0.152
SSL	0.056	0.487	0.083	-0.447	0.419	-0.217
T	0.424	-0.215	0.286	0.284	0.851	-0.051
DIAM	0.391	0.230	0.800	-0.069	0.285	-0.285
RBI	0.576	-0.151	0.885	0.039	0.534	0.168
RN	0.029	0.546	0.151	-0.376	0.072	-0.461
RP	0.315	0.269	0.378	-0.237	0.438	-0.209
RTD	0.000	-0.846	<0.001	0.786	0.344	0.253
SRA	0.002	0.721	0.001	-0.768	0.902	-0.033
SRL	0.111	0.414	0.029	-0.545	0.549	0.162

LA, leaf area; LAR, leaf area ratio; LC, leaf carbon content; LDMC, leaf dry matter content; LN, leaf nitrogen content; LP, leaf phosphorus content; SLA, specific leaf area; SSL, specific stem length; T, leaf thickness; DIAM, fine-root diameter; RBI, root branching intensity; RN, root nitrogen content; RP, root phosphorus content; RTD, root tissue density; SRA, specific root area; SRL, specific root length.

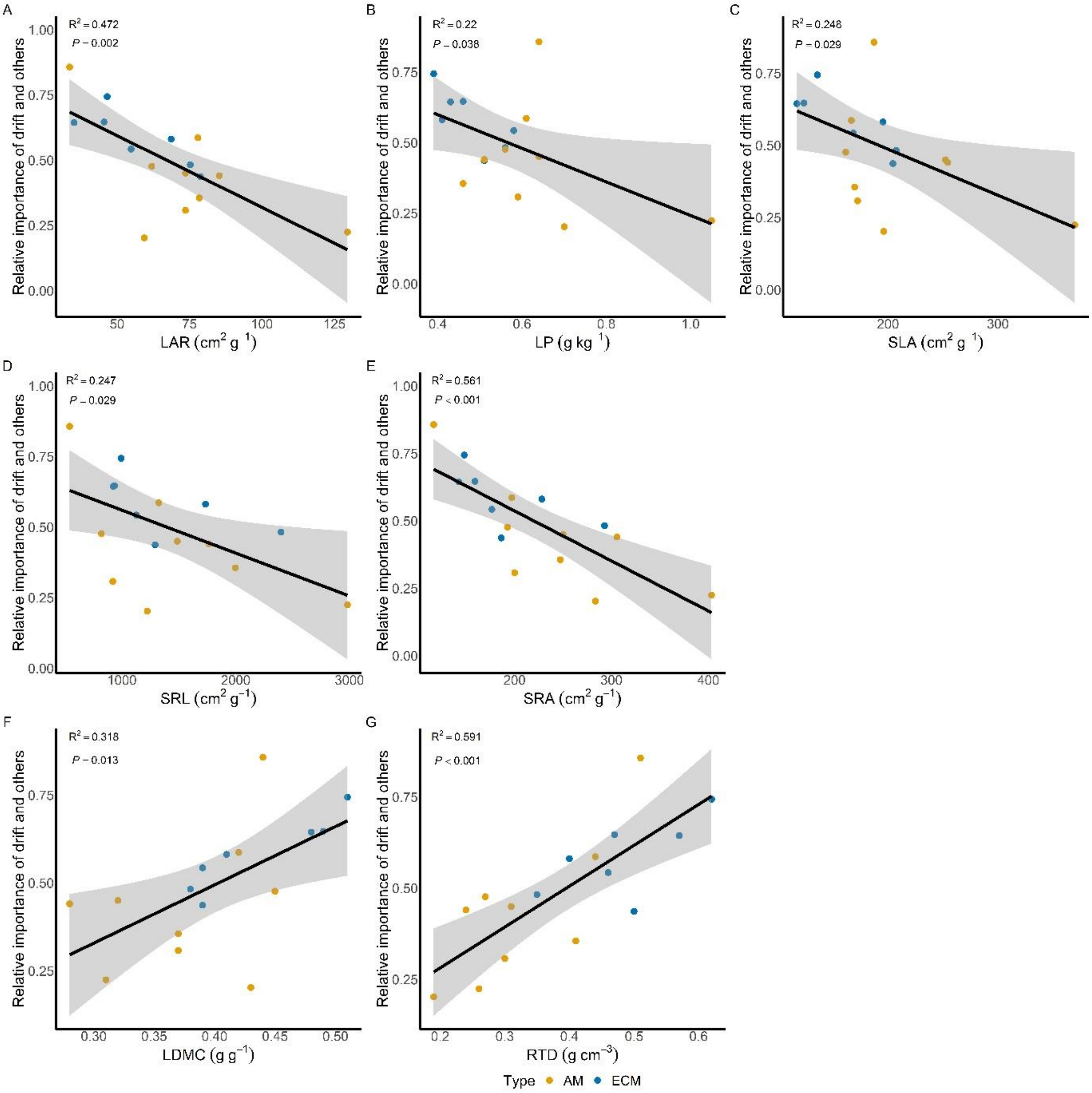

Figure S1 Effects of different functional traits on the relative importance of drift and others. LAR, LP, SLA, SRL and SRA are functional traits indicative of acquisitive resource-use strategy (A-E), while LDMC and RTD are functional traits indicative of conservative resource-use strategy (F-G). Solid lines represent regression lines with significant effect, and shaded areas represent 95% confidence intervals. LAR, leaf area ratio; LP, leaf phosphorus content; SLA, specific leaf area; SRL, specific root length; SRA, specific root area; LDMC, leaf dry matter content; RTD, root tissue density.

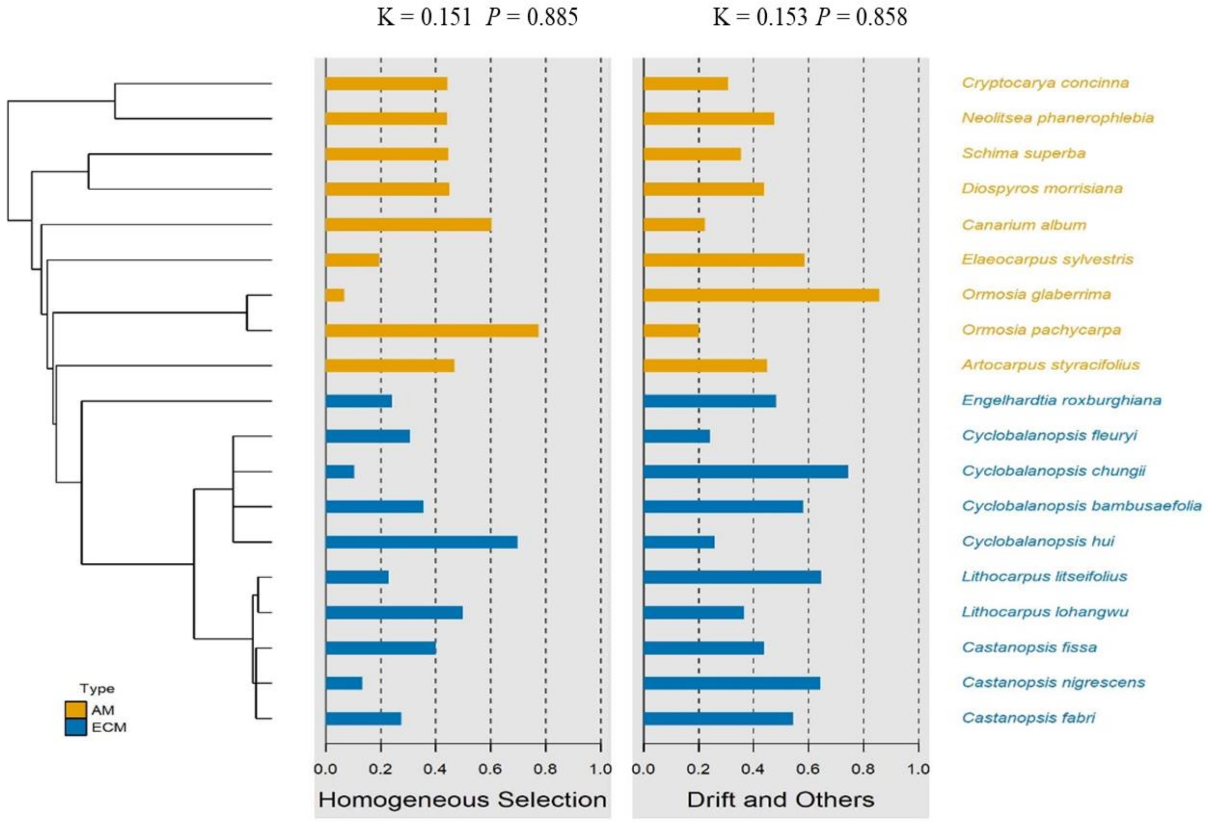

Figure S2 Phylogenetic signal analysis for the relative importance of homogeneous selection and drift (and others) in the root-associated fungal pathogen community of nineteen species.
